# Finding Relevant Psychoeducation Content for Adolescents Experiencing Symptoms of Depression: Content Analysis of User-Generated Online Texts

**DOI:** 10.2196/28765

**Published:** 2021-09-30

**Authors:** Kim K Dysthe, Ole R Haavet, Jan I Røssberg, Petter B Brandtzaeg, Asbjørn Følstad, Atle Klovning

**Affiliations:** 1 Department of General Practice/Family Medicine University of Oslo Oslo Norway; 2 Division of Psychiatric Treatment Research Department of Psychiatry University of Oslo Oslo Norway; 3 Department of Media and Communication University of Oslo Oslo Norway; 4 SINTEF Digital, Software and Service Innovation Oslo Norway

**Keywords:** adolescent, depression, internet, education, preventive psychiatry, early medical intervention, self-report, psychoeducation, information content, online, digital health, e-health

## Abstract

**Background:**

Symptoms of depression are frequent in youth and may develop into more severe mood disorders, suggesting interventions should take place during adolescence. However, young people tend not to share mental problems with friends, family, caregivers, or professionals. Many receive misleading information when searching the internet. Among several attempts to create mental health services for adolescents, technological information platforms based on psychoeducation show promising results. Such development rests on established theories and therapeutic models. To fulfill the therapeutic potential of psychoeducation in health technologies, we lack data-driven research on young peoples’ demand for information about depression.

**Objective:**

Our objective is to gain knowledge about what information is relevant to adolescents with symptoms of depression. From this knowledge, we can develop a population-specific psychoeducation for use in different technology platforms.

**Methods:**

We conducted a qualitative, constructivist-oriented content analysis of questions submitted by adolescents aged 16-20 years to an online public information service. A sample of 100 posts containing questions on depression were randomly selected from a total of 870. For analysis, we developed an a priori codebook from the main information topics of existing psychoeducational programs on youth depression. The distribution of topic prevalence in the total volume of posts containing questions on depression was calculated.

**Results:**

With a 95% confidence level and a ±9.2% margin of error, the distribution analysis revealed the following categories to be the most prevalent among adolescents seeking advice about depression: self-management (33%, 61/180), etiology (20%, 36/180), and therapy (20%, 36/180). Self-management concerned subcategories on coping in general and how to open to friends, family, and caregivers. The therapy topic concerned therapy options, prognosis, where to seek help, and how to open up to a professional. We also found young people dichotomizing therapy and self-management as opposite entities. The etiology topic concerned stressors and risk factors. The diagnosis category was less frequently referred to (9%, 17/180).

**Conclusions:**

Self-management, etiology, and therapy are the most prevalent categories among adolescents seeking advice about depression. Young people also dichotomize therapy and self-management as opposite entities. Future research should focus on measures to promote self-management, measures to stimulate expectations of self-efficacy, information about etiology, and information about diagnosis to improve self-monitoring skills, enhancing relapse prevention.

## Introduction

### Background

The highest loss of disability-adjusted life-years (DALYs) due to depression is seen in mid-adolescence [1]. Previous studies report a 5.6%-15% lifetime cumulative prevalence of major depressive disorder (MDD) in youth [2-4]. Recent epidemiologic studies measuring cumulative prevalence in shorter timespans reveal rates ranging from 2% to 17.7% [5-9]. Subthreshold depression and symptoms of depression are found in 9.3%-20% of adolescents [5,6,10], with studies revealing higher rates in girls [6]. The subsequent increased risk of early disability, adverse mental disorders, and suicide later in life [11-16] calls for intervention at adolescent age [17-21]. Although depression is frequent among adolescents, they also appear hesitant to talk openly about mental problems to family, friends, caregivers, and health personnel [22-24]. Instead, young people increasingly use social media, online influencers, and internet search engines to gather health information. Such information may frequently be misleading, not supported by clinical research, and believed to suppress help-seeking behavior [25]. When an intervention opportunity eventually arises, adolescents are inclined to drop out from therapy [26-28]. Consequently, we must consider new ways to design relevant and engaging clinical solutions to stimulate help-seeking behavior and promote therapy adherence [29]. Several attempts to develop such strategies have shown promising results [30].

In general, previous studies suggest that accurate initial information about depression through the realm of psychoeducation could promote help-seeking behavior and treatment compliance, self-management, and social functioning and may alter the course of depression [31,32].

We believe the established clinical principles of psychoeducation could be used to develop diverse technological solutions for youth mental health services [33,34]. However, psychoeducation is characterized by an extensive fauna of therapeutic approaches and theories, masking the efficacy of its various informational components [35,36]. To reveal its full potential as a prerequisite for clinical technologies, such as informational websites, online therapy, or chatbots, we need to develop population-specific psychoeducational content targeting adolescents with depression.

The results of this study could also be applied as a basis for a more extensive toolkit for clinical use in both primary and secondary health care. Psychoeducation is regarded as an integral part of different therapeutic strategies, such as cognitive behavioral therapy (CBT). In the future, we need this kind of research as a cornerstone to develop novel clinical programs based on the principals of joint clinical and technological care.

### Prior Work

According to previous studies, psychoeducation seems to be effective in treating depression, although as an integral component of more extensive therapy programs or fields [37-39]. In general, internet-based interventions for depression and anxiety disorders prove efficacious in adults [31,40]. Similar solutions also show promising results in adolescents [39,41,42]. Although 2 systematic reviews suggest that psychoeducation may prevent early onset and support management of adolescent depression [43,44], more research in this area is needed.

In the literature, the definition of psychoeducation varies. For instance, defining it as *different types of information about the disease, essential to the patient’s biological, psychological, and social function* [43,44] does not cover all its clinical goals. The boundaries between passive health information and generic therapeutic problem solving remain fuzzy. Instead, a more fruitful path may be to pursue the clinical *aims* of psychoeducational intervention: (1) to provide recipients with health-specific insight and (2) to enhance the level of self-efficacy [40].

First, to provide insight, psychoeducation could be provided passively on websites, in written materials, as technological solutions, as health information prior to further therapy, or in initial classroom lectures [34,43]. To meet this aim, professional consensus claims that health-specific insight should contain crucial elements of information topics about etiology, prevalence, symptoms and self-monitoring, treatment, natural course, and therapy prognosis [45,46].

The second overarching aim of stimulating self-efficacy builds on social learning theory [47,48]. According to this, self-efficacy is regarded as a necessity for self-management and rests on the perceived expectation that actions required for coping and self-preservation are obtainable. Following social learning theory, measures stimulating self-efficacy expectations are just as important for therapy as a sturdy working alliance.

To enhance the experience of self-efficacy, the most frequently used psychoeducational programs extend into the habitat of CBT [33,34,49,50] and interpersonal therapy [51,52]. Examples of 2 commonly used programs are the Interpersonal Psychotherapy program [51] and the multicomponent therapy course Adolescent Coping with Depression [53]. The first focuses on social and interpersonal skills as a road map to symptom relief. The latter developed from the original Coping with Depression course by focusing on information about pleasant activities and relaxation exercise, adding an extra section on parental support and family structure. Later, similar programs introduced elements of metacognitive therapy and positive psychology, as well as lessons about cognitive restructuring, identifying and altering negative beliefs and thought patterns [49].

Building on these 2 clinical aims, existing psychoeducation programs are originally developed from established theories and therapeutic models and their efficacy later tested in clinical trials. However, data-driven research aiming to improve our knowledge about what *content* of psychoeducation is relevant and engaging to adolescents suffering from depression remains severely limited [34,36,45,49,54].

We found only 2 previous studies aiming to fill this knowledge gap: (1) Bevan-Jones et al. [50] derived psychoeducational content from focus groups and semistructured interviews with young persons with or at risk of getting depression. The study also included parents, caregivers, and professional staff, suggesting attention toward mood versus symptoms of depression, possible reasons, self-management, and where to get help [50]. (2) Bru et al. [49] investigated the user experience of different components of a frequently used program derived from the Adolescent Coping with Depression course. In this study, the program participants found the initial general information about depression useful. Even if they found the latter part concerning cognitive restructuring and identification of thoughts difficult to use, it made them more able to regulate emotional reactions.

### Goal of This Study

Our study ultimately aims to specify what information topics regarding depression are relevant to young people. Contrary to the previous studies, we use the spontaneous nature of natural text data in 870 posts related to symptoms of depression on Norway’s largest public online information site for young people. To the best of our knowledge, our study will be the first to analyze unmoderated data revealing the information needs of adolescents with symptoms of depression, enabling us to answer the following research question: What information is most relevant to adolescents experiencing symptoms of depression?

This study will contribute knowledge about population-specific information needs, helping developers employ the full potential of psychoeducation for technology-based information platforms and clinical, technological solutions.

## Methods

### Study Design

In response to the research question and the large amount of text data, we conducted a content analysis [55] using Crabtree and Miller’s template approach. The method includes developing an a priori codebook from the findings of previous research or analyses. In this case, to answer the research question, the codebook consists of topics from existing psychoeducation programs. The analysis was chiefly deductive in nature. However, to avoid missing important information, we also inductively developed new codes to a refined codebook during the process of analysis. The choice of study design allowed us to gather insight into what adolescents regard as relevant, described in their own words in posts concerning depression submitted by youth to a public information service.

### Participants and Data

The service ung.no is the official youth information website provided by the Norwegian Directorate for Children, Youth and Family Affairs. The website is available for everyone, although Norwegian is the only language. The website’s target population is young people in the age range of 13-20 years. The editorial staff provides secure, reliable, and relevant information through online articles and multimedia content such as animated videos, alongside a question-and-answer (Q&A) section where young people can choose from a predefined list of topics, such as economy, education, work, family, social relations, and health.

Through a data transfer agreement between the project and the Norwegian Directorate for Children, Youth, and Family Affairs, we gained access to 277,552 anonymized posts written from the years 2005 to 2018 in Microsoft Excel format. The young people using the service tag their post with age, gender, and a main topic from a predefined drop-down list before submitting it to a staff of nurses, psychologists, and doctors. One of the topics is mental health and emotions*.* In the entire dataset, there were 14,804 posts belonging to this topic. When replying, the staff tags the questions with 1 or more categories within each main topic. Each post can contain more than 1 category, organized in a given order. One of them is the category *depression*.

**Figure 1:**
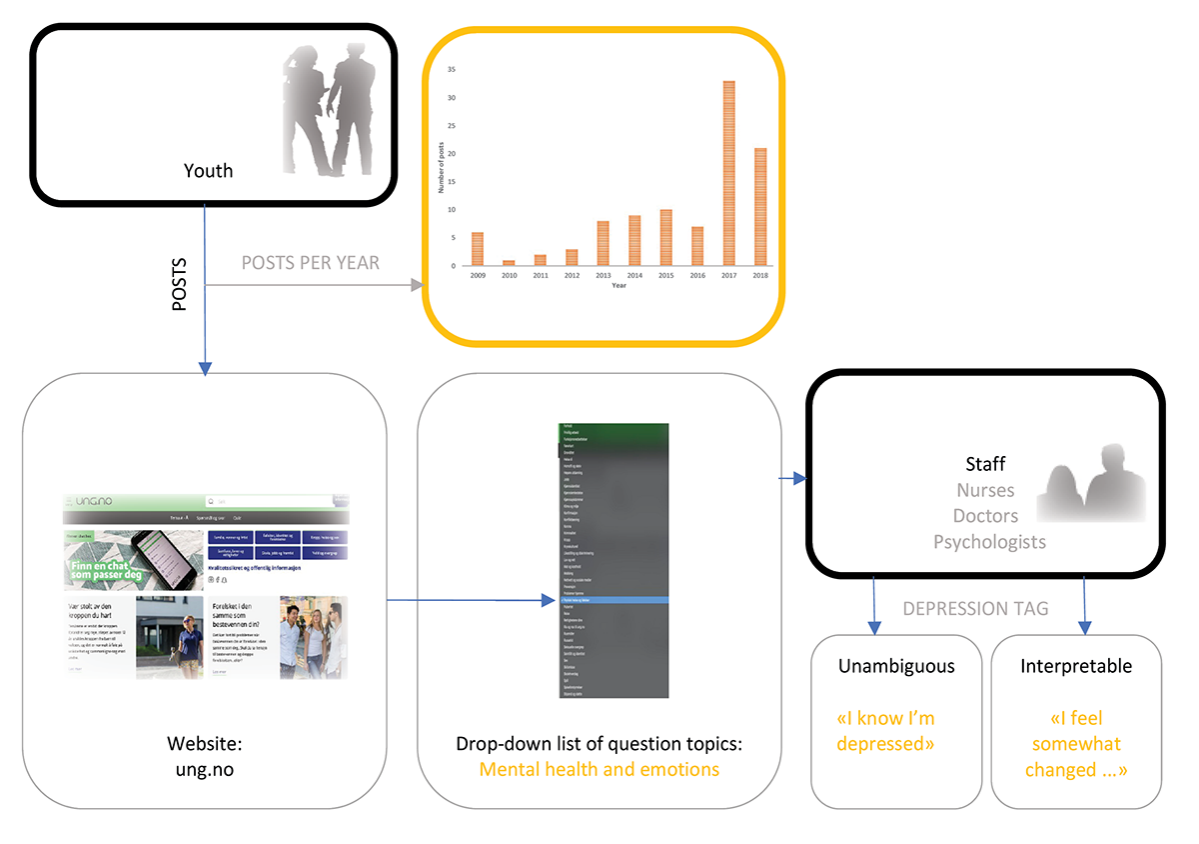
Data collection path.

Even though the data are preorganized and exported from SQL to Excel format, this specific data source is not commonly used in research. The number of depression posts written by people belonging to the age category of late adolescence (16-20 years) was 870. The posts were registered between the years 2007 to 2018, although most of the posts in the sample were written between 2013 and 2018 (see Figure 1).

To identify false negatives, that is, relevant posts not included in the initial categorization, we applied the Excel randomization function for a random selection of 150 posts from the entire dataset of 275,731 nondepression posts. We found only 3/150 (2%). A proportion (24%, 24/100) of the posts in the 100-post sample did not reveal any symptoms of depression, described other conditions such as bipolar disorders or personality issues, or portrayed someone else than the writer. We did not include these posts in the content analysis.

For further details about the data characteristics, refer to Table 1 and Figure 1. Some posts contained only a single specific question (see examples of posts in Textbox 1). Others conveyed a description of the situation and symptoms without any question at all. A few posts concerned third persons, such as friends or relatives, suffering from depression, seeking advice on how to relate. There was also some variation in the questions posted: Some of them unambiguously pointed to a specific topic. A significant proportion was polysemantic, disabling analysis based on computerized text mining, dependent on an interpretation of the post as a single unit of meaning.

Overall, the dataset provides unique insight into the information needs and beliefs about mental health during late adolescence and young adulthood. Analyzing this type of data has several advantages for developing technological or clinical programs specific for this group: First, we identify what information the target group demands when experiencing symptoms of depression, asking questions on public information websites, thereby demonstrating an information need. In contrast to data obtained through surveys or interviews, user-generated data from online services or social media are considered less influenced by the study context and environment, the relationship with an interviewing clinician, and the state of mood only at the time of assessment [56]. Second, the posts could describe narratives covering a longer time frame, providing a better opportunity for assessing cognitive processes. Moreover, the writers identify and describe their thoughts in their own words, at a time when they feel a need to express them, thus making the data valid for topic relevance valuation. For these reasons, online user-generated data may be recognized as an important tool for detecting and analyzing mechanisms of depression [57,58].

**Table 1:** Selected data features.

Data characteristics	Percentage and numbers
Posts included for content analysis	76
Posts revealing no symptoms of depression, other conditions, or describing someone else	Not analyzed: 24% (24/100)
Depression interpretation	Unambiguous: 59% (45/76) Interpretable: 41% (31/76)
Secondary health care level, when specified	18% (14/76)
Comorbidity described	34% (26/76)
Gender (entire sample, 100 posts)	Boys: 23% (22/97) Girls: 77% (75/97) Not specified: 3 posts
Age (entire sample, 100 posts)	16 years: 31% (31/100) 17 years: 24% (24/100) 18 years: 13% (13/100) 19 years: 22% (22/100) 20 years: 10% (10/100)
Mean word count per post (entire sample, 100 posts)	180 (17,997/100)

**Examples of posts, paraphrased before being translated to English.**

- “I'm so depressed I don’t want to live. My father beat my mom and me. I feel that people fail me. I’m sad. I’m sick and tired of not coping. I started high school, but I can’t keep up. My favorite school subjects aren’t interesting anymore. When I fail, I just give up. I cry a lot. I'm way too depressed to go to school. I'm in conflict with the others in my class, and cannot go to school when I feel bad, so I stay home. My friends are always together. But I feel like they'll shut me out. Now I'm no longer invited to things. I deleted everyone on Facebook, then they wondered why I don’t talk to them anymore. I feel even more sad and want to die. Nobody cares about me. I wonder if I can talk to my girlfriend about that, but I feel like she will not understand me. Everything I try is useless. I don’t quite know what to do anymore. I've talked to a counselor at school. He asks me to complete high school, but I can’t. I just want an honest answer about what to do without involving psychologists.”
- “Hi! I do not feel good enough, not pretty enough. I'm losing myself. I'm not who I once was. Before, I smiled and was never tired. Now I just want to die. I have suffered from depression since primary school. Lately it has actually been better. I have friends, family and a girlfriend who cares for me. I still just want to die. Recent weeks I have cried for nothing. I am thinking of taking my life and I'm a bad person to think this way, that I will leave all the good stuff behind. I have visited a psychologist but cannot open up. The psychologist only tells me things I already know. No therapists will take me seriously. I always smile and behave nicely, but I do not think it is wise, in fact, I've been thinking about hospitalization. I’m unstable. I can commit suicide any time. But if I am admitted, they will never listen anyway. I know that. They're just going to send me on to someone else who's talking about how to get better, just that, to get rid of my anxiety and depression, that simple. All this I know. I'm confused. All I know is that I want to die. That's why I write. What can I do with my life?”

### Procedures

We developed an a priori codebook (Table 2) from the information topics about the different psychoeducation programs [45,46,51-53,59], predefining the following upon analysis: etiology of depressive disorders, frequency, natural course, therapy prognosis, therapy options, and diagnosis. From the interpersonal and cognitive theories, we added the topics of self-management and social skills [49-51].

From the 870 posts, we randomly extracted 100 for further analysis using the Excel randomization function. Two raters, a general practitioner (author KKD) and a psychiatrist (author JIR), both possessing cognitive therapy education and training, coded the posts using the NVivo 12 Pro application for qualitative analysis.

**Table 2:** A priori codebook (8 codes).

Codes: questions	Description/beliefs
1. Etiology: Why is it like this?	Beliefs about causality
2. Frequency: Is it common?	Beliefs about the prevalence of depression
3. Course: How will it develop?	Beliefs about the natural course of depression
4. Prognosis: Will it work?	Beliefs about therapy efficacy
5. Therapy: Will therapy make me better, and where can I seek help?	Beliefs about therapy options
6. Diagnosis: What is wrong with me?	Beliefs about diagnosis
7. Self-management: What can I do myself?	Beliefs about coping
8. Social support and participation: How do I open, and where do I seek social support?	Beliefs about the importance of social support

**Figure 2:**
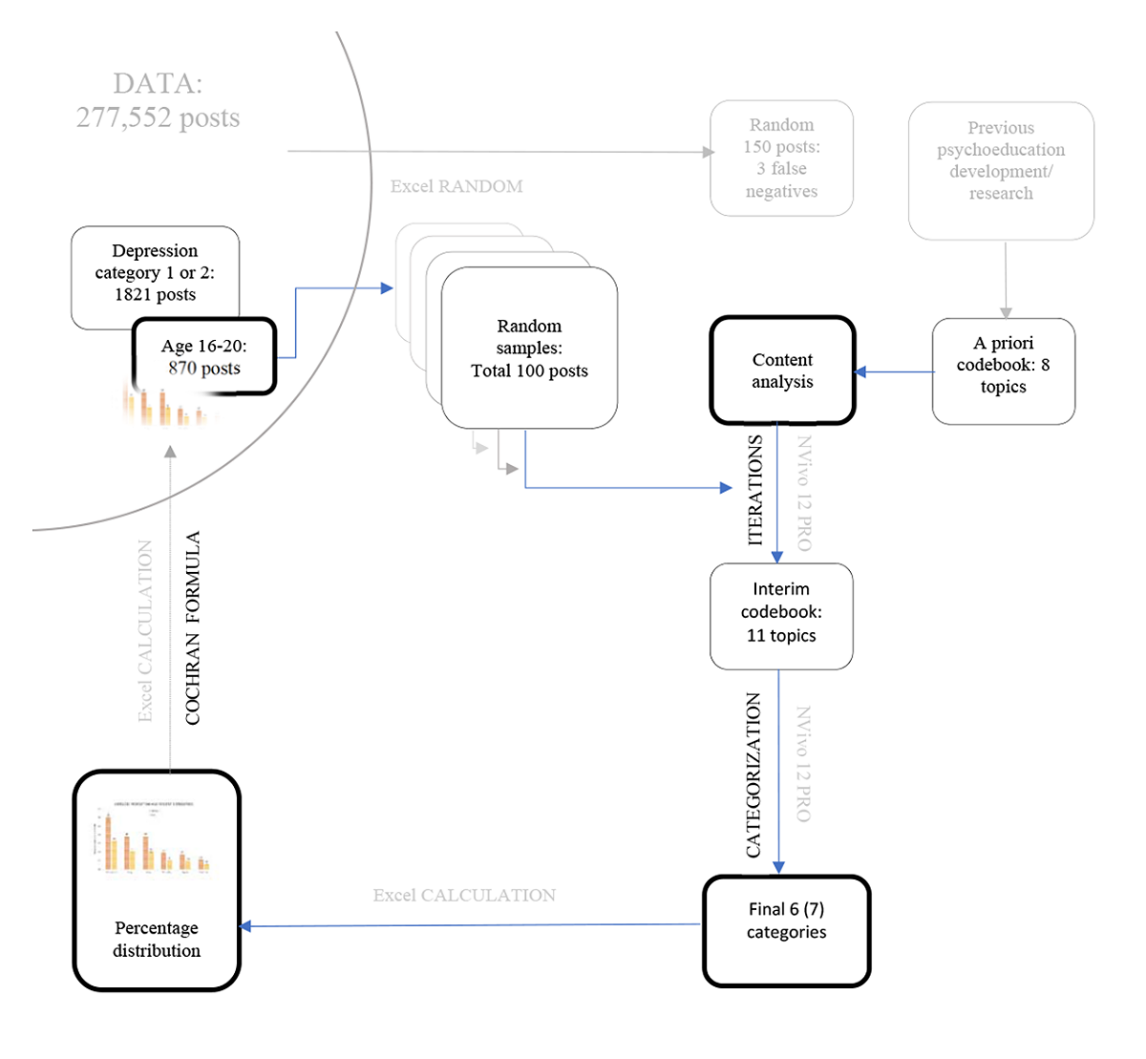
Analysis procedure from data selection to results.

As visualized in Figure 2, we coded both questions and descriptions into the same corresponding NVivo nodes. To avoid missing information, each rater established novel codes containing topics not described in the codebook. We performed 5 iterations. For every iteration, the 2 raters met to discuss the analyzing process. We developed subcodes for every topic and agreed upon a refined codebook after every meeting. After 34 posts, no new codes were added, leaving an interim codebook of 11 codes. We then used the NVivo 12 Pro function “Aggregate Coding from Children“ to calculate the reference count for each of the 11 interim codes and subcodes (Table 3). The interim codebook went through a final refinement, merging overlapping codes and subcodes into categories and subcategories due to similarity of content. The references were summarized into a total count for each category. Using Excel, we calculated each category’s reference count proportion within the 100 posts and the percentage distribution of categories in the total number of text references.

**Table 3:** Interim codebook and new codes with corresponding numbers of NVivo references (11 codes).

Code	Count	Classification
1. Etiology: Why is it like this? (Beliefs about causality)	35	Initial code
2. Frequency: Is it common? (Beliefs about the prevalence of depression)	1	Initial code
3. Course: How will it develop? (Beliefs about the natural course of depression)	12	Initial code
4. Prognosis: Will it work? (Beliefs about therapy efficacy)	10	Initial code
5. Therapy: What kind of therapy? (Beliefs about therapy options)	45	Initial code
6. Diagnosis: What is wrong with me? (Beliefs about diagnosis)	16	Initial code
7. Self-management: What can I do? (Beliefs about self-management)	33	Initial code
8. Social support and participation (beliefs about the importance of social support)	37	Initial code
9. Beliefs about consequences for their parents	7	New code
10. Beliefs about rights and privileges	7	New code
11. Is it normal? (Beliefs about the normality of symptoms)	9	New code

### Statistical Analysis

Applying the Cochran formula [60], we calculated the adequate sample size (*n*_0_) for an infinite population and then used the additional formula to calculate the sample size (*n*) of the finite population of 870 posts (*N*). Analyzing 100 posts enabled us to estimate the percentage distribution of each theme in the dataset of 870 posts with a 95% confidence level (*Z=*1.96) and a 9.2% ± margin of error (*e*), providing a 0.5 variance value (*P*=.5). For statistical comparison between the sample and the 870-post dataset, we included all the 100 posts as part of the sample, including those omitted from the content analysis (24%, 24/100).

### Reliability

Regarding reliability, the first rater (KKD) coded the posts. The second rater (JIR) then went through all the coded posts. In cases of disagreement, the second rater suggested alternative coding or a new code. We reviewed all alternative or new coding in a code meeting for every iteration and generated new codes in 20% (43/212) of the cases. In all, 3 of the codes remained unresolved. These 3 codes were omitted from the final analysis.

### Reflexivity

During the coding process, we discussed all codes that could reveal different interpretations. In numerous cases, both raters could relate connotations to real patients. Such topics were discussed to overcome biased interpretations. In most cases, the interpretation was straightforward, built on descriptions of common symptoms of depression. As far as possible, we strived to anchor the analysis on unambiguous functions of language, alongside a clear reference to previous research and theory.

## Results

### Preliminary Typology

Adding to the codebook (Table 3), we identified 3 new codes: questions and beliefs about normality, specific rights and privileges given to people with depression, and how depression will affect their parents.

As expected, the data revealed little overlap between the a priori codebook topics, leaving a single category for most of the codes. Based on social learning theory and text meaning, we interpret the actions of social support and participation closely related to the concept of self-efficacy. Hence, we merged the codes and references in the interim categories of self-management and social skills into a new self-management category. We did this for 2 reasons: First, social learning theory does not differentiate between the experience of self-efficacy obtained through actions related to social contact and other types of self-management, such as physical activities, jobs, and school achievements. Second, we also find this relation in the dataset revealing an expectation to manage; however, the adolescents seemed insecure about how. Consequently, ways of initiating social interaction and support are described as something they demand advice about in order to manage on their own, for instance, how to open up about their mental problems to friends and family. For the same reason, we added the new interim code *beliefs about consequences for their parents*, implying advice about involving parents, not only as a protective factor against suicidal and parasuicidal behavior, but also as a measure to support self-management.

We also merged the references to the codes *what kind of therapy* and *therapy prognosis* into the therapy category, due to the cognitive demand for information to alter expectations. In the posts, we found a temporal component in the questions about therapy, asking what to expect from a therapist or hospital admission, effects and side effects of medicines, and an overall demand for information about therapy prognosis in general.

Additionally, the data showed an essential issue in the polysemantic question “What should I do?” Although not frequently asked, the question reflects an important finding discussed later. This category was constructed from references coded to both *self-management* and *therapy* categories in the original a priori codebook and from one single reference from the social support code.

The 2 remaining new codes were much less referred to than the codes in the original codebook. We added 2 of them to the *miscellaneous* category.

### Estimated Category Proportion (p^) and Percentage Distribution

**Figure 3:**
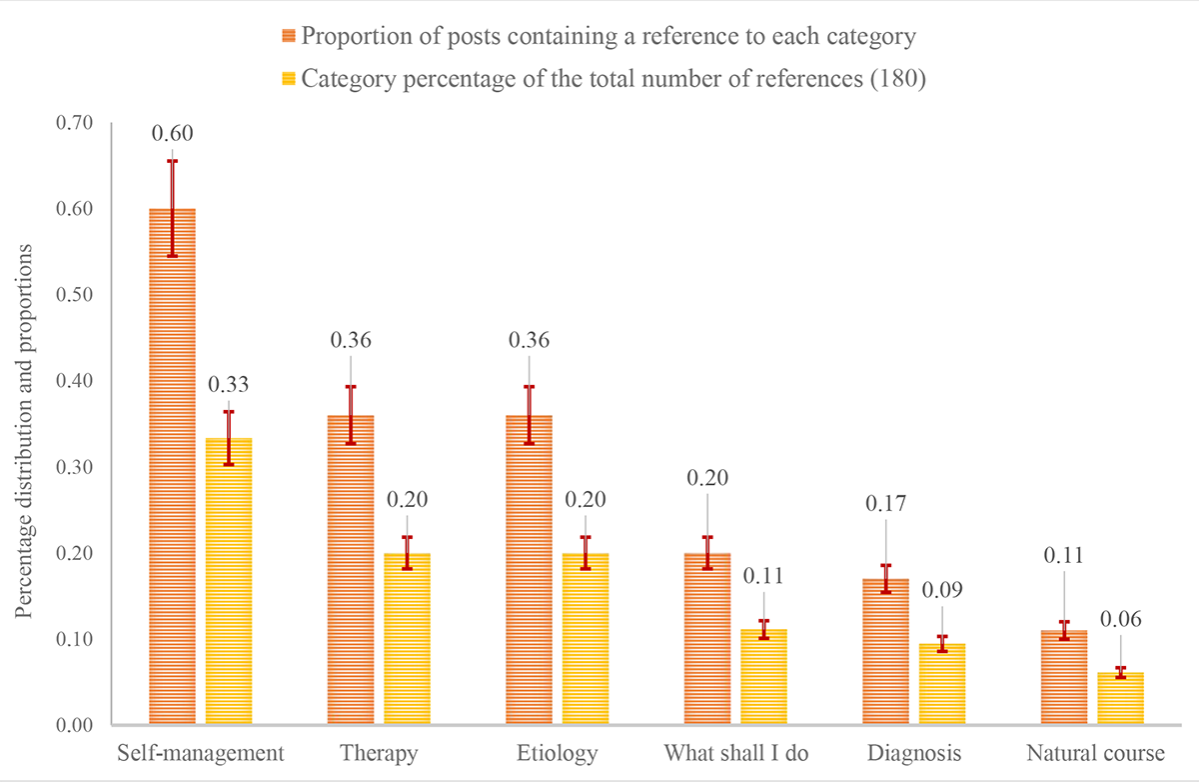
Proportion (p^) of posts containing references to each category. Percentage distribution of the total count of references, with error bars showing a ±9.2% margin of error.

In line with the category-constructing process, we applied NVivo 12 Pro to merge the corresponding codes and reference count with the different categories. We then calculated the estimated percentage distribution of the entire dataset of 870 posts, as presented in Figure 3: There was no overlap between the error bars of each column, meaning a ±9.2% margin of error will not affect the interpretation of the overall results. Analyzing a random sample of 100 posts was sufficient to answer the research question. The categories most frequently referred to were *self-management* (33%, 60/180), *therapy* (20%, 36/180), and *etiology* (20%, 36/180) (Table 4).

**Table 4:** Final category content and subcategories after coding and categorization.

Category	Count	Subcategories: beliefs and questions
1. Self-management	60	Coping in general How to open to friends and family Beliefs about consequences for their parents
2. Therapy	36	Therapy options Prognosis Where to seek help How to open up to a professional
3. Etiology	36	Risk factors Stressors Course of events leading to symptoms and behaviors
4. What should I do?	20	Manage on my own, as opposed to seeking professional help
5. Diagnosis	17	Symptoms conflicting with the self-concept The diagnosis defining the mood
6. Natural course	11	The course of depression as something out of control
7. Miscellaneous	11	Rights and entitlements when suffering from depression Normality regarding common psychological mechanisms

The *self-management* category (category 1) was most frequently referred to, revealing a demand for information about coping in general, how to open to friends and family for support, and how to relate to parents and caregivers. The *etiology* category (category 2) portrayed questions about risk factors and stressors leading to depression, as well as descriptions of events leading to symptoms and behaviors. In contrast, the *therapy* category (category 3) contained questions and beliefs about therapy options and therapy prognosis, where to seek professional aid, and how to open up to a professional regarding mental problems.

The category *What should I do?* (category 4) reflected the equivocalness of therapeutic interventions versus self-management. In a proportion of the posts, the narrative led to the question “What should I do?” This question occurred almost entirely in the context of the codes *what kind of treatment* or *self-management*. In several cases, this distinction was impossible to make, and these ambiguous text references were coded to both categories. One such reference that was coded to *social support and participation* also led to the same type of question. The posts regarding this topic described therapy, self-management, and social support either as different actions the writers believed could lead to improvement or as something they have already tried. Either way, the question did not belong to any of the a priori codes and revealed new beliefs about what actions to take when experiencing symptoms. Hence, we established a separate category for this question, counting each of the references coded to both *what kind of treatment* and *self-management* as one code in the *What should I do?* category (category 4).

The *diagnosis* category (category 5) consisted of questions and beliefs about what the symptoms could signify, describing experiences of being changed and demanding information about symptoms that resemble a disorder as something separate from the internal self-concept. At the same time, beliefs and questions about depression revealed a somewhat fatalistic view about the diagnosis as something defining mood without allowing variation, only susceptible to intervention. We found similar questions related to the *natural course* category*.* Beliefs about the course and prognosis of depression were regarded as something beyond control, relentlessly worsening the mental state, eventually resulting in suicidal thoughts.

Some of the new codes detected in the data were not frequently referred to; however, they were still worth mentioning due to a possible clinical significance. The code *What are my rights?* consisted of beliefs and questions about public financial support and absence from school and work when experiencing symptoms of depression. The code *Is it common?* consisted of questions and beliefs about disease prevalence, almost nonexistent in the data (count = 1). We generated a new code *Is it normal?* for questions and beliefs about the normality of psychological processes, complaints, and symptoms.

## Discussion

### Principal Results

We found that text references most frequently referred to the categories *self-management*, *etiology*, and *therapy*. The *therapy* category contains the subcategories of *therapy options* and *prognosis*, including where to seek initial help and how to relate to a health professional. However, measures to stimulate expectations of self-efficacy should also emphasize therapy as an active guiding process. Although the category of *etiology* was 1 of the most referred to, other categories, such as *diagnosis* and *natural course*, could possibly gain higher relative relevance later in the course of depression, when symptoms are less intrusive. This also applies to information about diagnosis, previously thought to improve self-monitoring skills aiming at preventing relapse.

### Limitations

Our data were most probably influenced by selection bias, as many of the posts were written by those with self-perceived severe or persistent symptoms of depression. When we investigated the information needs of adolescents experiencing symptoms of depression, we looked for the distribution of information topics among those asking questions on the online service. We had to bear this in mind when generalizing to a population level: These topics were determined relevant to adolescents with symptoms of depression asking for guidance. The information needs may not apply to every adolescent with depression. Rather, when in need of information, this is what they consider to be relevant. Simultaneously, we did not know the cognitive and emotional states of the adolescents submitting the posts. Conditions not apparent in the posts could affect data quality, for instance, drug abuse, personality disorders, social phobia, age-related mood swings, and other conditions commonly associated with unipolar depression, such as anxiety, bipolar disorders, and physical conditions (chronic pain, myalgic encephalomyelitis). Furthermore, categorizing the posts describing depression is only achievable according to descriptions of symptoms, not severity or persistence. The DSM-5 and ICD-10 diagnosis classification systems rate severity according to factors such as duration, loss of function, the presence of core symptoms, and the number of other symptoms. In this dataset, the only reliable hint toward severity assessment is the number of different depression symptoms presented in each post.

Even if the majority of the posts were written in the years 2013 to 2018, some posts were submitted in 2009 (see Figure 1). Young people’s language can change, possibly influencing the way they communicate issues about depression as a social construct. However, referring to the research question, we believe the experience of depression symptoms and the conjoining information needs are less influenced by changes in language over time. For example, the way young people regard common variations of mood as symptoms of a disease may be influenced by a proposed psychologization of youth language, nourished by education and social media. Nevertheless, referring to the previous literature, the content of psychoeducation is rooted in established theories of psychology and has not changed drastically, describing depression as a condition with biological presets. We believe the demand for specific information and the cognitive effect of such information, as described in existing psychoeducation development, to be consistent and less dependent on possible changes in language and attitudes.

### Comparison With Prior Work

Following the first principal aim of psychoeducation, previous development does not regard any of the information topics to be of superior importance. Nevertheless, we found in our study that psychoeducation should pay extra attention to some of the components, especially during the stages of depression where the level of symptoms is high. Since the topics of self-management and therapy are related to interventions and support, this kind of information is more relevant to adolescents when in despair. Regarding our second aim, that of strengthening self-efficacy, the high prevalence of reference to the *therapy* and *self-management* categories resonates not only with previous empirical research [45,49-51,53,61] but also with theory-driven development.

Interestingly, the principal results of this and other studies [45,49-51,53,61] could lead us to interpret social learning theory in a rather new perspective: Considering the spontaneous nature of self-reporting text data, our findings may indicate an inherent demand among these adolescents to manage on their own when experiencing symptoms of depression. Social learning theory describes self-efficacy as an expectation acquired through lifelong performance experiences [47]. Self-efficacy, the subsequent belief that different life challenges are manageable, is considered a chief ingredient for help-seeking and therapy motivation. This resonates with a central principle of CBT: the therapist as a guide to self-management.

When interpreting the content of the category *What should I do?*, we suggest that psychoeducation aim at enhancing the close relation between therapy and self-management, confirming the role of the therapist as actively underpinning performance skills [50]. In our study, we interpreted this question as a request for seeking advice about what to do in the current situation, referring to *either* exercises enhancing self-management *or* traditional therapy. This is not found in the 2 preceding studies. The category relates to the research question by conveying demands for guidance about actions to take when there are no apparent other options, revealing beliefs about these 2 types of interactions as opposite entities. Psychoeducation must also aim at overcoming the dichotomy of therapy versus self-management and provide information about the close integration of both.

Our study adds to previous research and psychoeducation development programs [45,50] on the questions and beliefs about *therapy*, as it contains the subcategories of *where to seek professional help* and *how to open up to a professional*. These are topics related to therapy, previously receiving little attention. Bevan-Jones et al. [50] found both young persons and professionals appreciating information about whom to contact for appropriate assistance. The *how to open up to a professional* subcategory reflects previous research about help seeking, suggesting that adolescents do need guidance not only to enhance social and familial openness and support but also to know where to seek initial professional help and what to expect from a therapist. In compliance with the clinical aim of preparing the patient for therapy [62], they require information about the role and communicating skills of a professional helper [22-24].

In our analysis of the questions categorized as concerning *etiology*, we found the adolescents to be particularly interested in risk factors and stressors (Table 3). We also found specific inquiries about etiology, summarized into the question *Why do I suffer from this?* Following previous psychoeducation development, alongside the findings of Bevan-Jones et al. [50], this question meets the aim of providing recipients with health-specific insight. It could also be that the adolescents refer to issues of causality as functions of language constituting an integral component of the narrative sequence of events leading to symptoms of depression. This could possibly explain the relatively high proportion of this topic.

In relation to the *diagnosis* category, CBT emphasizes the importance of self-monitoring skills [63]. Different psychoeducation programs for adolescents stress the ability to differentiate symptoms of depression from normal mood swings, thereby enabling teenagers with depression to seek earlier help and prevent relapse [45,50]. Although previous development regards the topic as highly important, we found a relatively modest attention directed toward symptoms of depression versus mood variations.

There seem to be mainly 2 ways to interpret this. First, as previously discussed, questions and beliefs about symptoms versus mood swings are relatively less relevant when experiencing symptoms. Second, this topic may have received less attention due to a lack of knowledge about the difference between age-related mood swings and symptoms of depression. Considering the self-reporting nature of the data, this could explain the low reference rates [34]. Regarding relapse prevention, we still believe self-monitoring abilities to be an important topic of psychoeducation and should be introduced to gain important insight into depression. It seems, however, more relevant to focus on relapse prevention when symptoms are less severe.

Considering the clinical implications and future research of our study, we believe this study can contribute to bringing more relevant and engaging psychoeducational content to the field of mental health information for adolescents and young adults. Initially, by reducing the risk of clinical information overload, we trust that the empirically derived information content could reduce program and therapy dropout and increase adherence when used in different technological solutions [24]. Then, by paying attention to content relevance, psychoeducation can focus on information about the disease rather than therapeutic techniques and theories, leading to the development of simpler and more efficient technological platforms and applications [64]. Future studies should focus on developing joint clinical and technological platforms targeting this population. Such research involves multiple steps, whereas deriving relevant information content is only the initial stage. Further research on machine learning technology, platform interface, and service design to enhance efficient integrations of data applications and clinical therapy is needed.

Beyond the scope of this study is also the analysis of specific cognitive patterns and relationships. We aim at deriving relevant information. At the same time, we lack empirical knowledge about the cognitive effects of such information. Using the same dataset, future analysis could let us investigate the information topics considering possible cognitive and metacognitive mechanisms and their ability to alter beliefs about depression. We also acknowledge a potential benefit in population- and disease-specific cognitive tools developed from such self-reporting text data. Text mining and machine learning analysis algorithms could be used on the entire dataset of all of the 14,804 posts regarding mental health to analyze combinations of symptoms and risk factors, alongside patterns of associated aspects, such as gender, age, social status, and comorbidity. From this kind of research, we may develop case-finding technological tools to alert clinicians about signs of depression in users of different e-mental health solutions. This kind of research could possibly enhance our understanding of which patients are best suited for different kinds of therapy.

Finally, additional research on psychoeducation information content using other sources of data is required. Previous studies have used focus groups or individual interviews to ask young people about information preferences undergoing treatment or participating in psychoeducation programs [49,50]. One study also involved the staff, therapists, and parents [50]. We suggest that future research on psychoeducational content be supplemented by using different sources of information. Considering the disadvantages of response bias in interview and focus group data and the shortcomings in data collection control using self-reporting observational data, survey studies gathering information from a broader spectrum of youth could be considered, for instance, from younger adolescents or young people without symptoms of depression. We also suggest supplementary studies using different sources of information from the internet and social media platforms using the advantages of participatory observation, such as active netnographic approaches. Studies such as this could possibly expand our apprehension of young people’s information needs and demands. The field requires a broader spectrum of research involving different methodologies, data sources, populations, and people contributing to the complex field of youth mental health.

### Conclusions

We analyzed user-generated online text data from adolescents with symptoms of depression to investigate what components of psychoeducation they regard as relevant. For clinical use, we suggest that developers of psychoeducational content and future research focus on the following:

- Measures to promote self-management and information about therapy options and prognosis, including where to seek initial help and how to relate to a health professional.
- Measures to stimulate expectations of self-efficacy and portray therapy as an active guiding process, thereby stimulating help-seeking behavior, motivation, and therapy adherence.
- Information about etiology.
- Information about diagnosis to improve self-monitoring skills and thus to enhance relapse prevention. Emphasis on this topic may be more relevant when symptoms are less severe.

## Abbreviations

CBT: cognitive behavioral therapy
DALY: disability-adjusted life-year
MDD: major depressive disorder
Q&A: question and answer
